# The Mereology of Depression—Networks of Depressive Symptoms during the Course of Psychotherapy

**DOI:** 10.3390/ijerph19127131

**Published:** 2022-06-10

**Authors:** Inken Höller, Dajana Schreiber, Fionneke Bos, Thomas Forkmann, Tobias Teismann, Jürgen Margraf

**Affiliations:** 1 Department of Clinical Psychology, University of Duisburg-Essen, 45141 Essen, Germany; dajana.rath@uni-due.de (D.S.); thomas.forkmann@uni-due.de (T.F.); 2 Department of Psychiatry, Interdisciplinary Center Psychopathology and Emotion Regulation (ICPE), University Medical Center Groningen, University of Groningen, 9700RB Groningen, The Netherlands; f.m.bos01@umcg.nl; 3 Psychiatric Hospital Mental Health Services Drenthe, Outpatient Clinics, 9401LA Assen, The Netherlands; 4 Mental Health Research and Treatment Center, Faculty of Psychology, Ruhr-Universität Bochum, 44787 Bochum, Germany; tobias.teismann@rub.de (T.T.); juergen.margraf@ruhr-uni-bochum.de (J.M.)

**Keywords:** network analysis, depression, BDI, CBT

## Abstract

(1) Background: Research has shown that it is important to examine depressive symptoms in the light of the mereology (the ratio between one symptom and the whole disorder). The goal of this study was to examine changes in the symptom interrelations of patients undergoing cognitive behavioral therapy treatment (CBT) via network analyses. (2) Method: Outpatients with depressive symptoms (*N* = 401) were assessed with the Beck Depression Inventory three times (pretreatment, after 12 sessions, and post-treatment) during CBT. Gaussian graphical models were used to estimate the relationships among symptoms. (3) Results: The severity of depressive symptoms significantly decreased over the course of therapy, but connectivity in the networks significantly increased. Communities of symptoms changed during treatment. The most central and predictable symptom was worthlessness at baseline and after 12 sessions, and loss of energy and self-dislike at post-treatment. (4) Conclusion: The results indicate that the severity of depressive symptoms decreased during cognitive behavior therapy, while network connectivity increased. Furthermore, the associations among symptoms and their centrality changed during the course of therapy. Future studies may investigate individual differences and their impact on the planning of psychotherapeutic treatment.

## 1. Introduction

Depression is one of the most prevalent mental disorders worldwide, with more than 264 million people suffering from it [1]. Considering that the burden of depression constantly rises globally [1], there is tremendous need for prevention to protect those who have not been affected yet, and treatment for those who already suffer.

So far, one of the most examined treatments for depression is cognitive behavioral therapy (CBT) [2]. A review of 16 meta-analyses of Butler, et al. [3] found not only a large effect size for CBT as a treatment for unipolar depression but also that CBT was superior to antidepressants in the treatment of adult depression. However, the evidence seems mixed. While Johnsen and Friborg [4] reported that the effects of CBT have seemed to decline linearly and steadily since its introduction; this was disconfirmed by Ljótsson, et al. [5] and Cristea, et al. [6]. A new network meta-analysis revealed no superiority of CBT over other treatments [7].

Given this mixed evidence regarding treatment efficacy and the risk of detrimental outcomes, the evaluation of the therapy outcomes (in diverse phases of psychotherapy) of patients is essential. There are several ways to evaluate therapy and the presence of depressive symptoms. Usually, treatment outcome is assessed by self-report questionnaires or structured interviews of depressive symptoms. One of these self-report measures is the *Beck Depression Inventory* (BDI-II) [8], which is the most common self-report measure used to assess the severity of depressive symptoms worldwide [9]. The BDI-II assesses the symptoms of depression beyond those stated in the ICD-10, since it is inclined towards the DSM-IV [10]. Normally, the sum score of the BDI-II is taken to reflect the presence or the severity of a depressive episode. However, focusing on only the sum scores obscures potentially relevant information about the symptoms themselves [11]. This was further substantiated by McGrath [12], who stated that terms such as depression and other mental disorders include conceptual complexity (e.g., cognitive and emotional impairment) that is ignored by simply building total scores of symptom complexes.

Recent research has therefore suggested moving away from the predominant view that mental disorders constitute latent constructs. Instead, it is suggested to examine the symptoms themselves and the relationships among them [13,14]. The network theory of psychopathology proposes that mental disorders arise not due to an underlying common cause, but because symptoms interact so that they eventually lead to a full-blown disorder [15]. To advance our understanding of how psychopathology changes over the course of treatment, it can be relevant to study the associations between depressive symptoms and not depression as a single construct [11]. One statistical approach to do this is network analysis, which estimates and visualizes the associations among individual symptoms [13,16,17].

The network theory has gained considerable popularity in recent years, as evidenced by a sharp increase in studies using network analysis [18]. One approach taken to operationalize the network theory is to examine the co-occurrence of symptoms on cross-sectional self-report questionnaires such as the BDI-II [17]. In these networks, the nodes are depressive symptoms, and the edges are the correlations among these symptoms. This approach allows us to study how symptom interrelations change over the course of treatment. Apart from the associations themselves, other network characteristics may also be insightful. For example, it has been suggested that symptoms may be more strongly connected in depressed individuals than in healthy individuals or remitted patients [19]. However, evidence for this hypothesis so far has been mixed in cross-sectional network studies [20], with several studies reporting increased connectivity in depressed individuals [21,22], whereas others reported the opposite, namely increased connectivity after antidepressant treatment [23,24]. Network analysis also offers the option to reveal which symptoms are particularly central in a symptom network. Such central symptoms are strongly connected to other symptoms in the network and have therefore been hypothesized to play a central role. Central symptoms that have often been identified in networks of depressive symptoms are sadness, anhedonia, energy loss, and guilt [20,21,25,26]. 

In summary, network analysis could advance our understanding of depression and the associations among depressive symptoms. Thus far, however, only a few studies have examined how symptom networks change between consecutive time points in the same patient group. Furthermore, no study has examined the changes in cross-sectional networks over the course of psychological depression treatment. The main goal of this study was, therefore, to compare the networks of depressive symptoms in a large sample of outpatients at three different stages of cognitive behavioral therapy (at the beginning, after 12 sessions, and the end of therapy). In more detail, we hypothesized that (1) the connectivity, (2) the community structure, (3) the centrality, and (4) the predictability of depressive symptoms assessed via the BDI-II in outpatients would differ before, after 12 sessions, and after CBT.

## 2. Materials and Methods

### 2.1. Sample

The sample comprised *n* = 401 outpatients treated at an outpatient psychotherapeutic clinic aged between 18 and 70 years (M = 36.4, SD = 13.1); 65.1% (*n* = 261) were female and 34.9% (*n* = 140) were male. The most common diagnoses according to the ICD-10 [27] were affective disorders (66.6%), whereby 202 participants (50.37%) were diagnosed with a unipolar depression as an ensured diagnosis. In total, 113 participants (28.18%) were diagnosed with anxiety, dissociative, stress-related, somatoform, and other non-psychotic mental disorders; 10 participants (2.49%) were diagnosed with behavioral syndromes associated with physiological disturbances and physical factors; eight participants (2.00%) were diagnosed with schizophrenia, schizotypal, delusional, and other non-mood psychotic disorders; and, last but not least, three participants (0.75%) were diagnosed with personality disorders. In total, 141 participants (35.16%) had at least one mental disorder comorbidity. The inclusion criteria were symptoms of depression, at least 18 years of age, and speaking German. Patients were excluded when they were under acute intoxication or experiencing acute psychotic symptoms. Prior to participating, all participants were informed about the research purpose of the data collection, the voluntary nature of their participation, data storage, and security. All participants gave written informed consent. The data collection procedure was in accordance with the Declaration of Helsinki [28,29] and approved by the Ethic Committee of the Ruhr-Universität Bochum (No. 318/2016).

### 2.2. Procedure

Participants received cognitive behavioral therapy (CBT) between December 2016 and September 2019 at the outpatient psychotherapeutic clinic of the Ruhr-Universität Bochum in Germany. All therapists received supervision every four sessions on average. The treatment relied on the manual for CBT of Hautzinger, et al. [30]. All participants who started therapy in this clinic were asked to complete the BDI-II. Participants were then assessed regularly during therapy; at the beginning of therapy, after four sessions, after 12 sessions, after 24 sessions, and after 40 sessions depending on the individual end of therapy. In the present study we focused on the BDI data before therapy (pre-CBT), after 12 sessions (12 sessions of CBT), and at the end of therapy (post-CBT). The length of therapy and the number of therapy sessions and thus the end of the treatment differed among participants. Sixty-three percent (*n* = 253) finished therapy after 24 sessions, 28.7% (*n* = 115) finished after 25 to 60 sessions, and 8.2% (*n* = 33) finished after 61 to 100 sessions. Participants who finished therapy after 24 sessions received short-term therapy, participants with 25 to 60 sessions received long-term therapy, and participants with 61 to 100 sessions received an extension of long-term therapy. Participants’ data were included in the study when they provided complete data for all three time points of data collection. If data were missing, the participants were excluded.

### 2.3. Measures

The BDI-II [8,31] is a self-report questionnaire that assesses the severity of depressive symptoms. The BDI-II consists of 21 items, each item representing one symptom (e.g., loss of pleasure, guilty feelings, agitation), which have to be rated on a 4-point Likert scale ranging from 0 to 3, with higher scores indicating greater severity of the symptom. Normally, a total sum score ranging from 0 to 63 is built, with higher scores reflecting a more severe depressive symptomatology overall. The German version of the BDI-II has been shown to be valid and reliable, with good internal consistency (Cronbach’s α = 0.84) [9]. We used the BDI-II to construct networks of its 21 individual items at each of the three time points. The distribution of all single items was illustrated in histograms (see Figure S1 in the Supplementary Materials).

### 2.4. Statistical Analyses

Networks were estimated at each of the three time points (pre-CBT, 12 sessions, and post-CBT). To ensure all networks consisted of the same sample, only complete data were used (missing data were not imputed). All networks therefore consist of *N* = 401 individuals. For all statistical analyses, R version 4.0.1 was used.

To estimate networks of depressive symptoms, we computed Gaussian graphical models (GGMs). For visualizing all three networks of depressive symptoms (pre-CBT, after 12 sessions, and post-CBT), the R-package *qgraph* [16] was used. The visualization includes nodes representing depressive symptoms of the BDI-II and edges representing between-person cross-sectional associations among symptoms, estimated by partial correlations between the symptoms [16]. To reduce false positive edges, we applied the least absolute shrinkage and selection operator (Lasso) [32]. For the arrangement of symptoms in the networks, the Fruchterman–Reingold algorithm [33] was used, placing nodes (symptoms) that were more strongly connected closer to each other and nodes that were less or not at all connected further apart. The layouts of the networks were averaged to allow visual comparison of the symptom networks before CBT, after 12 sessions of CBT, and after the CBT.

#### 2.4.1. Network Connectivity

For each network, network connectivity was calculated by summing all the absolute edge weights. Differences in network connectivity before CBT, after 12 sessions, and after CBT were compared using the network comparison test in the R package *nct* [34]. This test uses permutation tests to test differences in network connectivity for significance.

#### 2.4.2. Community Structure

Through exploratory graph analysis, we analyzed which nodes clustered together and may be considered to be part of the same underlying dimension. Communities were estimated with a random walk algorithm (walktrap) [35].

#### 2.4.3. Symptom Centrality

For each node in the network, strength centrality was computed by summing the absolute values of the edges from a given node to other nodes [36]. To examine the stability of strength centrality and to test the strength centrality among nodes for significance at each of the time points, we calculated the correlation stability coefficient (CS-coefficient) based on bootstrapping (*N* = 1000) using the R package *bootnet* [37]. The higher the CS-coefficient, the more reliable the interpretation of the order of centrality. The coefficients should not be below 0.25 and should preferably be above 0.5. 

#### 2.4.4. Predictability

Finally, we examined the predictability of nodes: how much the variance of a node is explained by its neighbors in the network [38]. This was calculated with the R-package *mgm* [39], with the scores ranging from 0 (the node cannot be predicted by other nodes) to 1 (the node can be perfectly predicted by others).

## 3. Results

### 3.1. Means and Variation of all BDI-II Symptoms

BDI sum scores decreased significantly from before CBT (22.4) to 12 sessions of CBT (17.4; t(400) = 11.33, *p* < 0.001), and from 12 sessions of CBT (17.4) to after CBT (13.0; t(400) = 9.3, *p* < 0.001). All individual item scores of the BDI-II also decreased significantly (see Table 1), except for punishment feelings (12 sessions to post-CBT), change in appetite (pre-CBT to 12 sessions), and loss of interest in sex (pre-CBT to 12 sessions). Regarding the standard deviations, from pre-CBT to 12 sessions, the variance over all BDI-II items did not change significantly from pre-CBT to 12 sessions and from 12 sessions to post-CBT.

### 3.2. Network Connectivity

**Figure 1:**
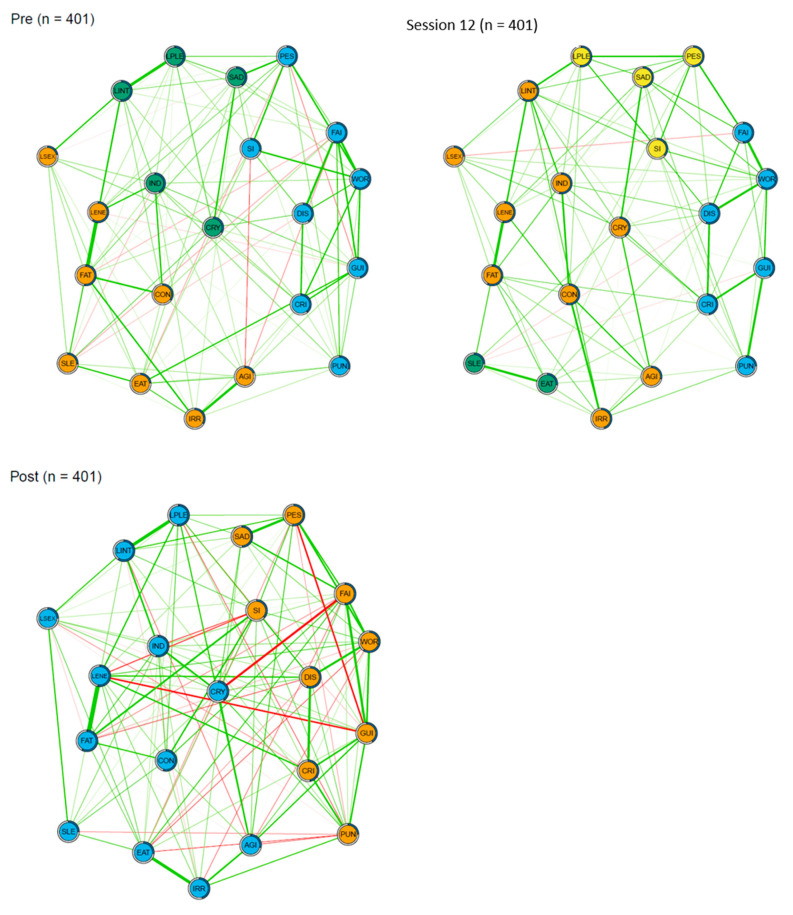
EBIC gLasso networks of BDI symptoms before CBT, after 12 sessions of CBT, and after CBT (*n* = 401). *Note.* Abbreviations are listed in Table 1. Green lines represent positive associations and thicker lines represent stronger associations between symptoms. The colors of the nodes represent different communities. Blue rings around the nodes represent the proportion of explained variance of that symptom.

The three networks of BDI-II symptoms before CBT, after 12 sessions of CBT, and after CBT are illustrated in Figure 1. The post-CBT network showed the highest connectivity: the sum of all edges was 14.0 compared with before CBT (10.3, *p* = 0.007) and with after 12 sessions of CBT (9.7, *p* = 0.011). The connectivity of the pre-CBT network and the network after 12 sessions of CBT did not differ significantly (*p* = 0.227).

### 3.3. Community Structure

Before CBT, three symptom communities were found (see Figure 1): (1) bodily symptoms (orange nodes) such as changes in sleeping and eating patterns, fatigue, and loss of energy; (2) affective symptoms (green nodes) such as sadness, crying, and loss of pleasure; and (3) cognitive symptoms (blue nodes) such as self-criticalness, self-dislike, and guilt. 

After 12 sessions of CBT, we found four communities: (1) (mostly) bodily symptoms (orange nodes) combined with crying, indecisiveness, and loss of interest, but not (2) sleeping and eating changes (green nodes); (3) (mostly) affective symptoms (yellow nodes) as well as suicidal ideation and pessimism; and (4) cognitive symptoms (blue nodes). 

After CBT, we found only two communities: (1) (mostly) affective–bodily symptoms (blue nodes) including sleeping and eating patterns and loss of pleasure, and (2) (mostly) cognitive symptoms (orange nodes).

### 3.4. Symptom Centrality

**Figure 2:**
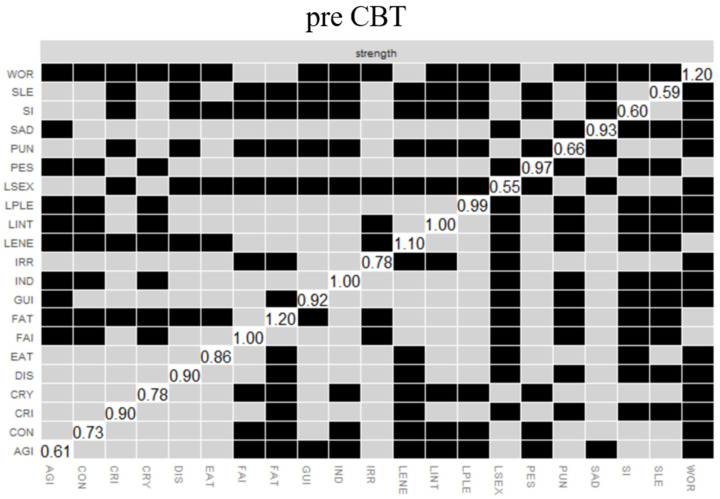

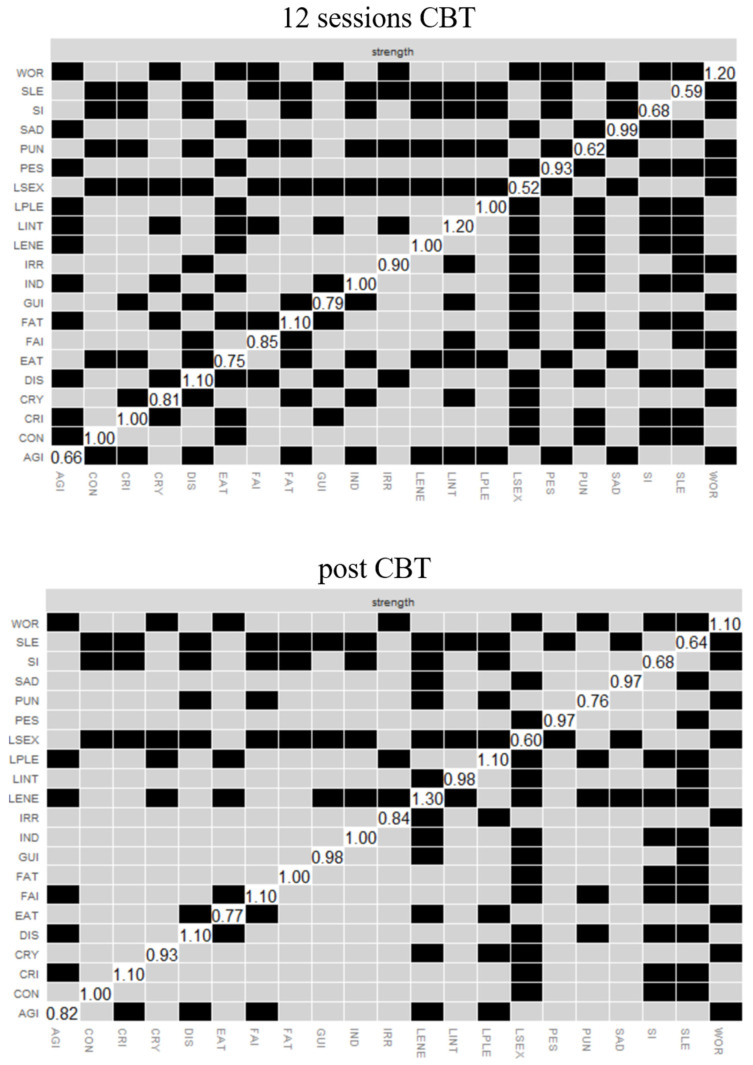
Bootstrapped difference tests of strength between nodes for the pre-CBT network, the network after 12 sessions of CBT, and the post-CBT network (*n* = 401). *Note.* Black boxes indicate nodes that differ significantly from one another, while gray boxes indicate nodes that did not differ significantly from one another. White boxes show the value of strength centrality for a given node.

Figure 2 illustrates the bootstrapped difference tests of strength between nodes in the pre-CBT network, the network after 12 sessions of CBT, and the post-CBT network (*n* = 401). Before CBT, two symptoms emerged as the most central: worthlessness, with a higher strength centrality than 16 other symptoms, and fatigue, with a higher strength centrality than 12 other symptoms. Other central symptoms were loss of energy, past failure, and pessimism. Loss of interest in sex, on the other hand, was the least central symptom in terms of strength, being significantly less central than 14 other symptoms in the pre-CBT network (see Figure 2, pre-CBT). The mean severity of depressive symptoms was not significantly associated with centrality (*r* = 0.43, *p* = 0.054), suggesting that the more central symptoms were not necessarily the more severe symptoms.

After 12 sessions of CBT, worthlessness remained the most central symptom, together with loss of interest. These were significantly more central than 11 and 10 other symptoms, respectively (see Figure 2, 12 sessions of CBT). Again, loss of interest in sex was the least central, which was significantly less central than 15 other symptoms in the network. After 12 sessions of CBT, centrality remained unassociated with the severity of depressive symptoms (*r* = 0.34, *p* = 0.126).

After CBT, loss of energy was the most central symptom, being significantly more central than 12 other symptoms in the network (see Figure 2, post-CBT). Again, loss of interest in sex was the least central symptom, which was significantly less central than 14 other symptoms in the network. Further, centrality remained unassociated with the severity of depressive symptoms post CBT (*r* = −0.14, *p* = 0.556).

The correlation stability coefficients were 0.594 for before CBT, 0.673 for 12 sessions of CBT, and 0.594 for after CBT, indicating at least a moderately reliable ordering of the centrality estimates, which should not be below 0.25 and should preferably be above 0.50 [37].

### 3.5. Predictability

Predictability indicates how much the variance of a node is explained by its neighbor in the network. Overall, the proportion of explained variance of all symptoms increased significantly from 0.39 (pre-CBT) to 0.44 (12 sessions of CBT, *t*(20) = −3.34, *p* < 0.01), and to 0.49 (post-CBT, *t*(20) = −3.57, *p* < 0.01). Before CBT, the greatest predictability (amount of variance of a node explained by its neighbors in the network) was found for worthlessness and loss of energy. After 12 sessions of CBT, loss of interest and worthlessness were the most predictable, and after CBT, loss of energy and self-dislike had the largest predictability. The predictability of the networks before CBT and after 12 sessions of CBT were correlated at 0.84 (*p* < 0.001), and that for the networks after 12 sessions of CBT and after CBT correlated at 0.89 (*p* < 0.001), indicating that if a node had high predictability before CBT, it also tends to have high predictability at 12 sessions and after CBT. 

Moreover, centrality and predictability were highly correlated before CBT (*r* = 0.82, *p* < 0.001) and after 12 sessions of CBT (*r* = 0.94, *p* < 0.001). In other words, symptoms that are strongly connected with other symptoms also seem to be strongly predicted by other symptoms before CBT and after 12 sessions of CBT. However, after CBT, this correlation became nonsignificant (*r* = 0.41, *p* = 0.064). While predictability was related to the mean severity of depressive symptoms before CBT (*r* = 0.59, *p* < 0.01), the correlations were nonsignificant after 12 sessions of CBT (*r* = 0.39, *p* = 0.078) and after CBT (*r* = 0.34, *p* = 0.132).

## 4. Discussion

Network analyses offer an exciting opportunity for a deeper understanding of how depressive symptoms co-occur, and how these change in the course of treatment. The main goal of this study was to examine networks of depressive symptoms and to compare those symptom networks at different stages of CBT (at the beginning, after 12 sessions, and at the end of CBT). 

The results of this study revealed that the connectivity among depressive symptoms increased over the course of therapy, whereas the severity of depressive symptomatology decreased. This finding is contrary to the hypothesis postulated by the network theory, in which symptoms are suggested to be more strongly connected in depressed individuals [19], as found by several empirical studies that compared the cross-sectional networks of patients with persistent vs. remitting depressive symptoms [21] and networks of patients treated with antidepressants [22]. However, our findings correspond to studies reporting stronger correlations between symptoms after therapeutic treatment [40] and those reporting increased network connectivity after antidepressant therapy [23,24]. These differing findings have been previously suggested to be due to increasing variance as the depressive symptomatology of the group decreases [20,23]. However, in the present study, the variance did not increase significantly overall, and only increased for one BDI-II item, making this explanation less likely. Another possible explanation for the conflicting evidence of studies is that studies in line with the connectivity hypothesis compared cross-sectional networks between groups (e.g., remitters versus persisters, or depressed individuals versus healthy controls), whereas studies with contrasting findings compared the same individuals at different time points. 

Although increased connectivity with decreasing symptom severity seems to be rather surprising from a network perspective, associations between different types of symptoms (e.g., cognitive and affective symptoms) are frequently addressed in CBT by differentiating among cognitive, affective, bodily, and behavioral aspects. This may have an impact on perceiving the relationships between different types of symptoms more consciously. Repetitively analyzing symptom cascades (trigger → cognition → affect → bodily symptom → behavior) might provide a deeper understanding of one’s own individual symptom patterns in typical situations causing depressive feelings. Unfortunately, the lack of a control group precludes us from attributing increased connectivity solely to the CBT. Furthermore, the associations depicted here are between-person, and may have limited generalizability to individual patients [41,42]. Future studies should focus on the influence of analyzing symptom cascades on individual networks.

Second, and as reported by Bos, Fried, Hollon, Bringmann, Dimidjian, DeRubeis and Bockting [23], communities changed from before therapy to after therapy. In the present study, three communities could be identified before CBT, including bodily, cognitive, and affective symptoms, whereas one more community emerged after 12 sessions of CBT, consisting of changes in sleeping and eating patterns. After CBT, depressive symptoms clustered into two communities: (mostly) affective–bodily and (mostly) cognitive symptoms. Intriguingly, a meta-analysis by Huang and Chen [43] also supported this two-factor solution for the BDI-II, with almost the same factor loadings as we found for the post-CBT assessment. Regarding temporal invariance of the factor structure over time, as an important psychometric requirement [44], previous studies reported contradicting findings, e.g., [23,40,45]. An important aspect might be that different studies used different measures for depressive symptomatology, and the different symptoms being assessed might also result in a different number of factors. Speculatively, we would expect that SSRI treatment [23] exerts a different impact on symptoms than CBT, especially when it comes to the cognitive evaluation of these symptoms. Additionally, the timeframe differed among studies, whereas previous studies [23,45] compared networks at baseline with networks at six weeks to two years, we examined networks after 12 sessions and at the end of therapy. Theoretically, it seems to be difficult to explain why communities should decrease after therapy. Similar to the findings on connectivity, this could be due to confounding factors. Fried et al. (2016) summarized the literature on the temporal measurement invariance of depression scales and detected a pattern: the factor structure might depend on symptom severity, with decreasing symptomatology resulting in fewer factors [45].

Third, the centrality of symptoms changed slightly over the course of therapy. While worthlessness and fatigue were the most central symptoms in the network at baseline and after 12 sessions, loss of energy seemed to be the most central symptom at the end of CBT. In addition, there seemed to be a relatively stable cluster of the most central symptoms, which have also been reported as most central in previous studies, including worthlessness (e.g., [23,26]), fatigue (e.g., [46]), loss of energy [21,23,25,26], and past failure (e.g., [26]). In a review of network studies in the field of depression by [20], positive affect or anhedonia were consistently identified as central nodes. However, comparing centralities from different studies is only informative to a certain extent, since we should keep in mind that centralities strongly depend on the symptoms being included in the network, as well as on the measures being used for centrality. This finding seems to fit the prior findings of increasing connectivity and decreasing communities at the end of CBT: increased associations (i.e., more and higher coefficients for the associations) among symptoms over the course of therapy lead to increased centralities (an increasing number of more central symptoms) and a stronger clustering of the symptoms and, hence, to fewer communities. Moreover, some symptoms, such as self-dislike and concentration, seemed to become more central during the course of therapy, whereas some, such as sleep as in [23,26] and suicidal ideation (as in Boschloo et al., 2016 [46]) did not seem to be central at any point of CBT in terms of their associations with other depressive symptoms. However, it should be noted that there has been a discussion on what centrality measures actually mean and that they are likely unsuitable as a measure for the causal impact of these symptoms on other symptoms in the network [47].

### 4.1. Practical Implications

Even though the meaning of the central symptoms in a psychological network has been discussed [47], the fact that some symptoms seem to be more strongly related to all other symptoms in the network should be further examined in clinical practice. Interestingly, symptom centrality was only moderately associated with symptom severity at the beginning and not at all associated with symptom severity at the end of CBT, meaning that more severe symptoms were not necessarily the most central ones. It might be possible that at the beginning of CBT, particular symptoms such as worthlessness, which was the most central but not the most severe, cannot yet be reflected or even detected by the patient. From clinical experience, it is easier for patients to report body-related symptoms at the beginning because these can be perceived more easily. During the course of CBT, cognitive and affective symptoms become more accessible for the patient because they are trained to perceive and differentiate among emotions, cognitive dysfunctions (such as fundamental assumptions of being worthless), and body sensations in CBT. Furthermore, symptoms that are (statistically) central in the network due to their relationships with other symptoms may not be as central in the patient’s perception. Therapy may benefit from an additional (statistical) perspective to add to the perceptions of patients and therapists of topics to address in treatment. The basis to do so would be person-specific networks.

For clinical practice, person-specific networks may provide interesting insights into symptom associations for individual patients [48]. Such networks can be constructed using, for example, data gathered through Ecological Momentary Assessment (EMA). EMA is a method used to repeatedly assess the symptoms of patients within minutes to hours via their smartphone [49,50,51]. EMA offers the option to assess the symptoms of patients in their daily lives, in the moment rather than retrospectively, providing valuable information on the course of symptoms over time and avoiding memory bias. Subsequent network analysis of EMA data might provide psychotherapists with interesting information on relevant associations among the daily life experiences of individual patients. Future studies should focus on the inter-individual variability of symptom networks and their individuality for patients with the same diagnosis. Furthermore, research should establish whether such person-specific networks improve the outcome of psychotherapeutic treatment and, if so, under which conditions. The use of such person-specific networks in therapy would join the movement of precision medicine and treatment selection in depression [52].

On the other hand, we should be cautious about not addressing symptoms in CBT due to their statistically low centrality in a network. Not addressing suicidal ideation, for instance, which constantly showed low centrality for all three networks, could have fatal consequences and would violate therapeutic guidelines. 

### 4.2. Strengths and Limitations

Strengths of this study include the relatively large sample (*N* = 401) and the use of state-of-the-art network techniques to examine changes in depressive symptom networks at different stages of CBT. Furthermore, we included a diverse sample of patients with mild to severe depressive symptoms, thereby covering the whole range of depressive symptomatology, as is common in an outpatient psychotherapeutic context. This strengthens the generalizability of our findings.

On the other hand, our findings should be interpreted in light of some limitations. Our data show rather low variance (as illustrated in the histograms) and a left-skewed distribution for individual items, especially for the suicidal ideation item. This may have influenced the results [53]. Nevertheless, we decided to include this item based on theoretical interest and clinical importance. Another limitation pertains to cross-sectional networks in general: the associations between nodes in these networks are between-person, which means we must interpret the uncovered associations as undirected and non-causal. It is also currently unclear whether between-person associations can be generalized to the individual patient [41,54]. It also has to be noted that we did not assess the racial/ethnic identification or the socioeconomic status of participants, which also might limit the results. Finally, due to the lack of a control group, we were unable to attribute any differences in the network structure directly to CBT.

## 5. Conclusions

The present study compared the networks of three consecutive time points before, after 12 sessions, and after CBT. The results indicated that all individual depressive symptoms decreased over the course of CBT. Furthermore, the network structure of depressive symptoms changed: network connectivity increased, and different symptom communities and central symptoms were uncovered. For future studies, it would be interesting to examine individual differences and their impact on the planning of psychotherapeutic treatment.

## Figures and Tables

**Table 1:** Means and standard deviations for all 21 BDI items.

No.	Item	Pre-CBT			12 Sessions of CBT			Post-CBT			Comparisons of *p*-Values			
		M	SD	Var	M	SD	Var	M	SD	Var	Pre vs. 12		12 vs. Post	
											M	Var	M	Var
1	Sadness	1.06	0.74	0.55	0.88	0.71	0.51	0.68	0.67	0.45	***	n.s.	***	***
2	Pessimism	1.02	0.94	0.89	0.70	0.87	0.76	0.49	0.79	0.62	***	**	***	***
3	Past failure	1.35	0.98	0.96	1.11	0.92	0.85	0.87	0.90	0.81	***	**	***	n.s.
4	Loss of pleasure	1.39	0.85	0.72	1.04	0.85	0.72	0.76	0.80	0.64	***	n.s.	***	***
5	Guilty feelings	1.06	0.86	0.74	0.90	0.80	0.64	0.69	0.78	0.61	***	**	***	n.s.
6	Punishment feelings	0.78	1.10	1.21	0.57	0.93	0.86	0.48	0.88	0.77	***	***	n.s..	***
7	Self-dislike	1.09	0.93	0.86	0.83	0.93	0.86	0.58	0.85	0.72	***	n.s.	***	***
8	Self-criticalness	1.21	0.92	0.85	0.95	0.92	0.85	0.73	0.90	0.81	***	n.s.	***	n.s.
9	Suicidal ideation	0.40	0.57	0.32	0.32	0.55	0.30	0.23	0.50	0.25	***	n.s.	***	***
10	Crying	1.03	1.05	1.10	0.70	0.99	0.98	0.52	0.91	0.83	***	*	***	***
11	Agitation	0.85	0.78	0.61	0.65	0.80	0.64	0.45	0.71	0.50	***	n.s.	***	***
12	Loss of interest	1.06	1.00	1.00	0.68	0.82	0.67	0.52	0.79	0.62	***	***	***	*
13	Indecisiveness	1.28	1.03	1.06	0.93	0.95	0.90	0.67	0.92	0.85	***	***	***	*
14	Worthlessness	1.23	1.03	1.06	0.91	1.02	1.04	0.70	0.93	0.86	***	n.s.	***	***
15	Loss of energy	1.21	0.77	0.59	0.98	0.74	0.55	0.68	0.75	0.56	***	n.s.	***	n.s.
16	Change in sleep	1.28	0.94	0.88	1.06	0.93	0.86	0.84	0.84	0.71	***	n.s.	***	***
17	Irritability	0.92	0.88	0.77	0.71	0.83	0.69	0.51	0.78	0.61	***	*	***	***
18	Change in appetite	0.83	1.00	1.00	0.75	0.96	0.92	0.59	0.86	0.74	n.s.	n.s.	**	**
19	Concentration difficulty	1.29	0.86	0.74	1.03	0.86	0.74	0.74	0.85	0.72	***	n.s.	***	n.s.
20	Fatigue	1.29	0.84	0.71	0.97	0.78	0.61	0.72	0.74	0.55	***	**	***	***
21	Loss of interest in sex	0.84	1.08	1.17	0.75	1.00	1.00	0.60	0.96	0.92	n.s.	**	**	**
	**BDI sum score**	22.44	12.02	144.48	17.40	12.03	144.72	13.03	11.82	139.71	***	n.s.	***	n.s.

Note. M = mean; SD = standard deviation; VAR = variance. The alpha was Bonferroni–Holm corrected for comparisons of the means; the Pitman–Morgan test was used to compare variances. * *p* ≤ 0.05, ** *p* ≤ 0.01, *** *p* ≤ 0.001, n.s. = not significant.

## Data Availability

All relevant data are reported within the article and are available from the corresponding author upon reasonable request.

## Supplementary Materials

The following supporting information can be downloaded at: https://www.mdpi.com/article/10.3390/ijerph19127131/s1. Figure S1: Histograms of each of the BDI items.

## References

[1] World Health Organization Depression. https://www.who.int/news-room/fact-sheets/detail/depression.

[2] Tolin D.F. (2010). Is cognitive–behavioral therapy more effective than other therapies?: A meta-analytic review. Clin. Psychol. Rev..

[3] Butler A.C., Chapman J.E., Forman E.M., Beck A.T. (2006). The empirical status of cognitive-behavioral therapy: A review of meta-analyses. Clin. Psychol. Rev..

[4] Johnsen T.J., Friborg O. (2015). The effects of cognitive behavioral therapy as an anti-depressive treatment is falling: A meta-analysis. Psychol. Bull..

[5] Ljótsson B., Hedman E., Mattsson S., Andersson E. (2017). The effects of cognitive–behavioral therapy for depression are not falling: A re-analysis of Johnsen and Friborg (2015). Psychol. Bull..

[6] Cristea I.A., Stefan S., Karyotaki E., David D., Hollon S.D., Cuijpers P. (2017). The effects of cognitive behavioral therapy are not systematically falling: A revision of Johnsen and Friborg (2015). Psychol. Bull..

[7] Cuijpers P., Quero S., Noma H., Ciharova M., Miguel C., Karyotaki E., Cipriani A., Cristea I.A., Furukawa T.A. (2021). Psychotherapies for depression: A network meta-analysis covering efficacy, acceptability and long-term outcomes of all main treatment types. World Psychiatry Off. J. World Psychiatr. Assoc..

[8] Beck A.T., Steer R.A., Brown G.K. (1996). Manual for the Beck Depression Inventory.

[9] Kühner C., Bürger C., Keller F., Hautzinger M. (2007). Reliabilität und Validität des revidierten Beck-Depressionsinventars (BDI-II). Der Nervenarzt.

[10] Frances A., First M.B., Pincus H.A. (1995). DSM-IV Guidebook.

[11] Fried E.I., Nesse R.M. (2015). Depression sum-scores don’t add up: Why analyzing specific depression symptoms is essential. BMC Med..

[12] McGrath R.E. (2005). Conceptual complexity and construct validity. J. Personal. Assess..

[13] Cramer A.O., Waldorp L.J., Van Der Maas H.L., Borsboom D. (2010). Comorbidity: A network perspective. Behav. Brain Sci..

[14] Markus K.A. (2008). Hypothesis formulation, model interpretation, and model equivalence: Implications of a mereological causal interpretation of structural equation models. Multivar. Behav. Res..

[15] Borsboom D. (2017). A network theory of mental disorders. World Psychiatry Off. J. World Psychiatr. Assoc..

[16] Epskamp S., Cramer A.O., Waldorp L.J., Schmittmann V.D., Borsboom D. (2012). qgraph: Network visualizations of relationships in psychometric data. J. Stat. Softw..

[17] Borsboom D., Deserno M.K., Rhemtulla M., Epskamp S., Fried E.I., McNally R.J., Robinaugh D.J., Perugini M., Dalege J., Costantini G. (2021). Network analysis of multivariate data in psychological science. Nat. Rev. Methods Primers.

[18] Robinaugh D.J., Hoekstra R.H., Toner E.R., Borsboom D. (2020). The network approach to psychopathology: A review of the literature 2008–2018 and an agenda for future research. Psychol. Med..

[19] Cramer A.O., Van Borkulo C.D., Giltay E.J., Van Der Maas H.L., Kendler K.S., Scheffer M., Borsboom D. (2016). Major depression as a complex dynamic system. PLoS ONE.

[20] Wichers M., Riese H., Hodges T.M., Snippe E., Bos F.M. (2021). A Narrative Review of Network Studies in Depression: What Different Methodological Approaches Tell Us About Depression. Front. Psychiatry.

[21] van Borkulo C.D., Boschloo L., Borsboom D., Penninx B.W., Waldorp L.J., Schoevers R.A. (2015). Association of symptom network structure with the course of depression. JAMA Psychiatry.

[22] Madhoo M., Levine S.Z. (2016). Network analysis of the Quick Inventory of Depressive Symptomatology: Reanalysis of the STAR* D clinical trial. Eur. Neuropsychopharmacol..

[23] Bos F.M., Fried E.I., Hollon S.D., Bringmann L.F., Dimidjian S., DeRubeis R.J., Bockting C.L. (2018). Cross-sectional networks of depressive symptoms before and after antidepressant medication treatment. Soc. Psychiatry Psychiatr. Epidemiol..

[24] Berlim M.T., Richard-Devantoy S., Dos Santos N.R., Turecki G. (2020). The network structure of core depressive symptom-domains in major depressive disorder following antidepressant treatment: A randomized clinical trial. Psychol. Med..

[25] Fried E.I., Epskamp S., Nesse R.M., Tuerlinckx F., Borsboom D. (2016). What are ’good’ depression symptoms? Comparing the centrality of DSM and non-DSM symptoms of depression in a network analysis. J. Affect. Disord..

[26] Bringmann L.F., Lemmens L., Huibers M., Borsboom D., Tuerlinckx F. (2015). Revealing the dynamic network structure of the Beck Depression Inventory-II. Psychol. Med..

[27] Dilling H., Mombour M., Schmidt M., Schulte-Markwort E. (2016). WHO: ICD-10 Kapitel V (F) Diagnostische Kriterien für Forschung und Praxis.

[28] World Medical Association (2001). World Medical Association Declaration of Helsinki. Ethical principles for medical research involving human subjects. Bull. World Health Organ..

[29] Williams J.R. (2008). The Declaration of Helsinki and public health. Bull. World Health Organ..

[30] Hautzinger M., Stark W., Treiber R. (2008). Kognitive Verhaltenstherapie bei Depressionen.

[31] Hautzinger M., Keller F., Kühner C. (2006). Beck Depressions-Inventar Revision: Manual.

[32] Tibshirani R. (1996). Regression shrinkage and selection via the lasso. J. R. Stat. Soc. Ser. B (Methodol.).

[33] Fruchterman T.M., Reingold E.M. (1991). Graph drawing by force-directed placement. Softw. Pract. Exp..

[34] Van Borkulo C.D., Boschloo L., Kossakowski J., Tio P., Schoevers R.A., Borsboom D., Waldorp L.J. (2022). Comparing network structures on three aspects: A permutation test. Psychol. Methods.

[35] Golino H.F., Epskamp S. (2017). Exploratory graph analysis: A new approach for estimating the number of dimensions in psychological research. PLoS ONE.

[36] Opsahl T., Agneessens F., Skvoretz J. (2010). Node centrality in weighted networks: Generalizing degree and shortest paths. Soc. Netw..

[37] Epskamp S., Borsboom D., Fried E.I. (2018). Estimating psychological networks and their accuracy: A tutorial paper. Behav. Res. Methods.

[38] Haslbeck J., Fried E.I. (2017). How predictable are symptoms in psychopathological networks? A reanalysis of 18 published datasets. Psychol. Med..

[39] Haslbeck J., Waldorp L.J. (2015). Structure estimation for mixed graphical models in high-dimensional data. arXiv.

[40] Fokkema M., Smits N., Kelderman H., Cuijpers P. (2013). Response shifts in mental health interventions: An illustration of longitudinal measurement invariance. Psychol. Assess..

[41] Bos E.H., Wanders R.B. (2016). Group-level symptom networks in depression. JAMA Psychiatry.

[42] Fisher A.J., Medaglia J.D., Jeronimus B.F. (2018). Lack of group-to-individual generalizability is a threat to human subjects research. Proc. Natl. Acad. Sci. USA.

[43] Huang C., Chen J.-H. (2015). Meta-analysis of the factor structures of the Beck Depression Inventory–II. Assessment.

[44] Widaman K.F., Ferrer E., Conger R.D. (2010). Factorial invariance within longitudinal structural equation models: Measuring the same construct across time. Child Dev. Perspect..

[45] Fried E.I., van Borkulo C.D., Epskamp S., Schoevers R.A., Tuerlinckx F., Borsboom D. (2016). Measuring depression over time... Or not? Lack of unidimensionality and longitudinal measurement invariance in four common rating scales of depression. Psychol. Assess..

[46] Boschloo L., van Borkulo C.D., Borsboom D., Schoevers R.A. (2016). A prospective study on how symptoms in a network predict the onset of depression. Psychother. Psychosom..

[47] Bringmann L.F., Elmer T., Epskamp S., Krause R.W., Schoch D., Wichers M., Wigman J.T., Snippe E. (2019). What do centrality measures measure in psychological networks?. J. Abnorm. Psychol..

[48] von Klipstein L., Riese H., Servaas M.N., Schoevers R.A. (2020). Using person-specific networks in psychotherapy: Challenges, limitations, and how we could use them anyway. BMC Med..

[49] Trull T.J., Ebner-Priemer U. (2014). The role of ambulatory assessment in psychological science. Curr. Dir. Psychol. Sci..

[50] Trull T.J., Ebner-Priemer U.W. (2009). Using experience sampling methods/ecological momentary assessment (ESM/EMA) in clinical assessment and clinical research: Introduction to the special section. Psychol. Assess..

[51] Santangelo P., Bohus M., Ebner-Priemer U.W. (2014). Ecological momentary assessment in borderline personality disorder: A review of recent findings and methodological challenges. J. Personal. Disord..

[52] Cohen Z.D., DeRubeis R.J. (2018). Treatment Selection in Depression. Annu. Rev. Clin. Psychol..

[53] Terluin B., De Boer M.R., De Vet H.C. (2016). Differences in connection strength between mental symptoms might be explained by differences in variance: Reanalysis of network data did not confirm staging. PLoS ONE.

[54] Bos F.M., Snippe E., de Vos S., Hartmann J.A., Simons C.J., van der Krieke L., de Jonge P., Wichers M. (2017). Can we jump from cross-sectional to dynamic interpretations of networks implications for the network perspective in psychiatry. Psychother. Psychosom..

