# Supplementary figures and images for: The Mereology of Depression—Networks of Depressive Symptoms during the Course of Psychotherapy
Source: Int J Environ Res Public Health. 2022 Jun 10;19(12):7131. doi: 10.3390/ijerph19127131 (PMC9222343; doi:10.3390/ijerph19127131)

## Supplementary material

**Figure S1.** Histograms of each of the BDI items.

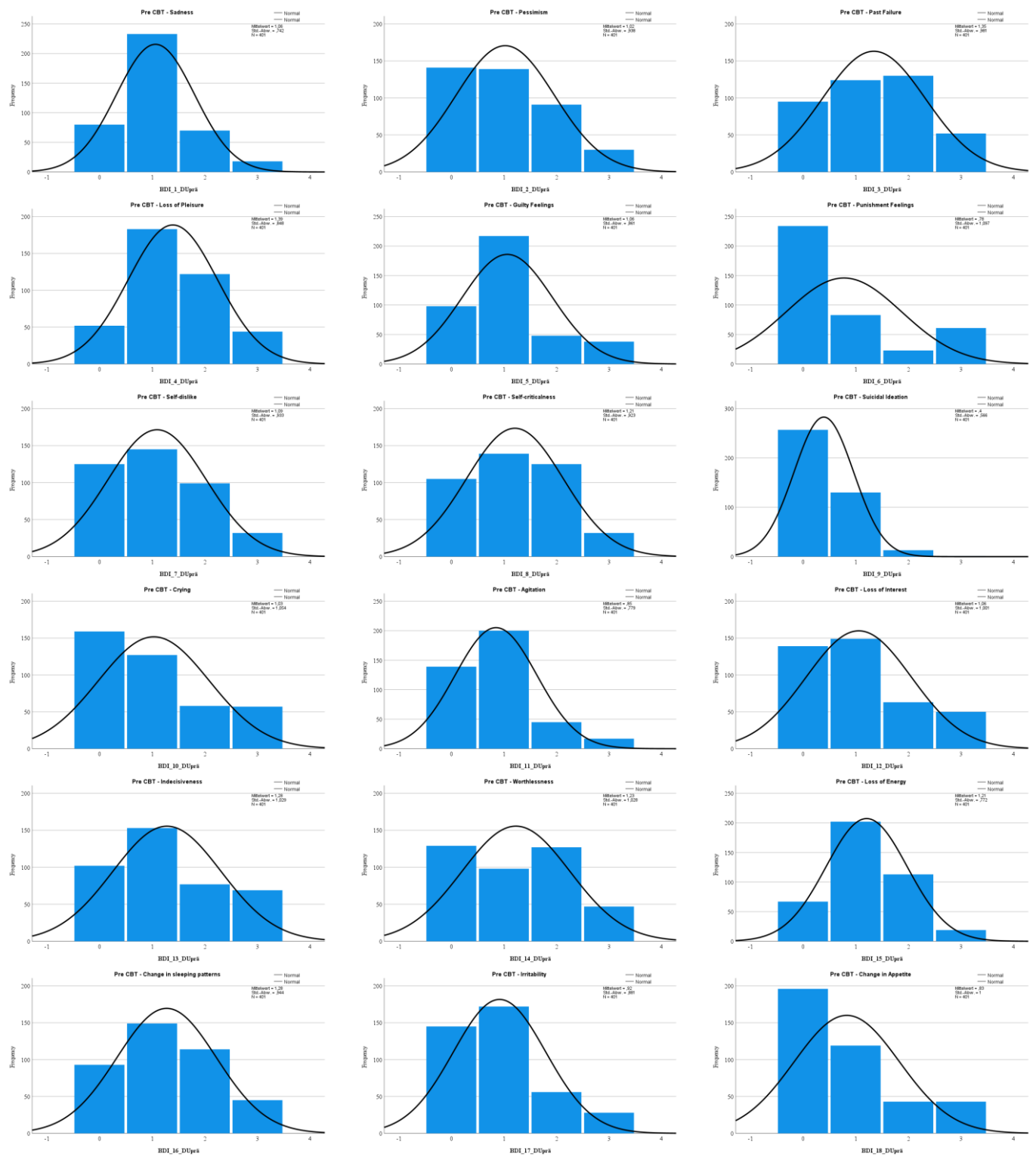

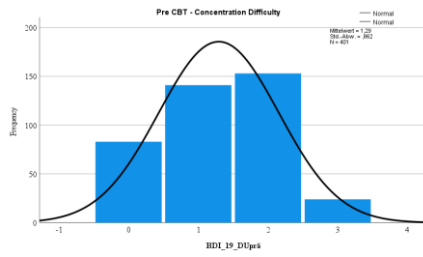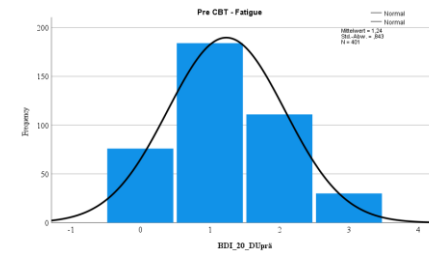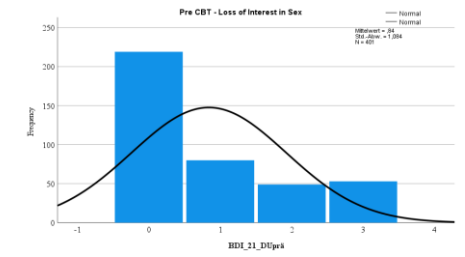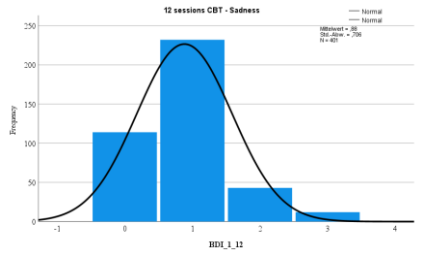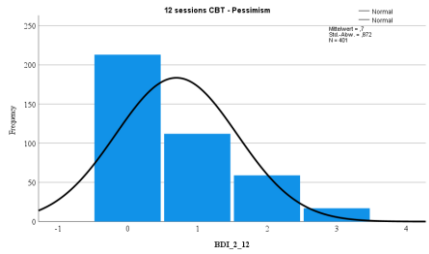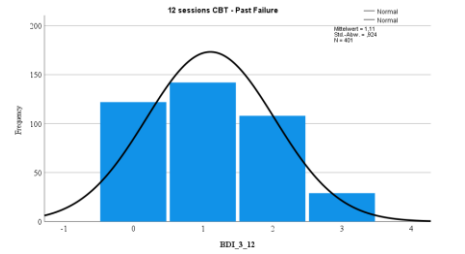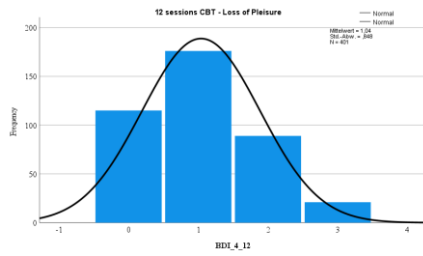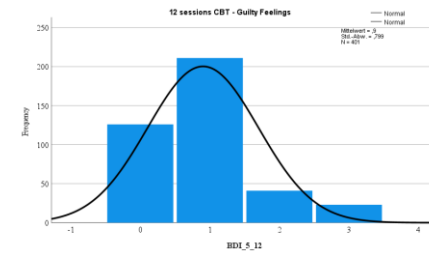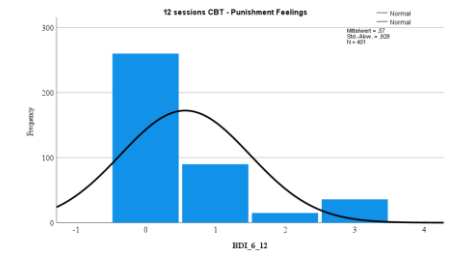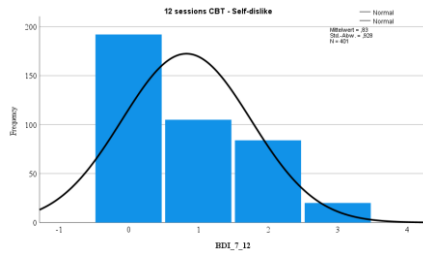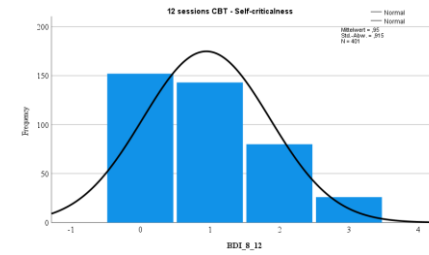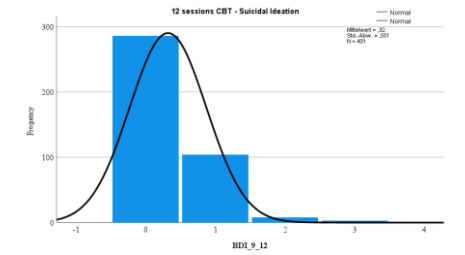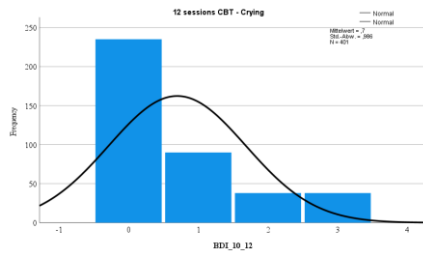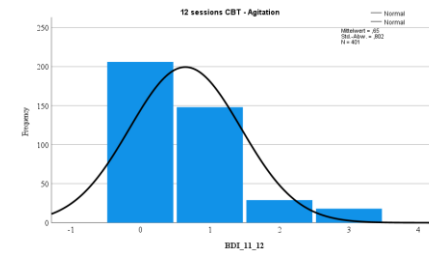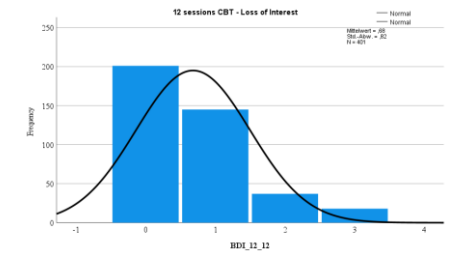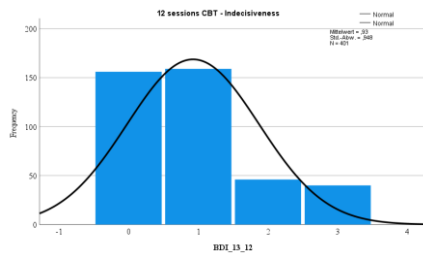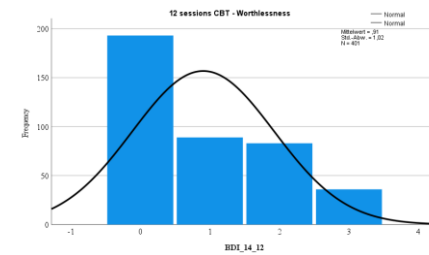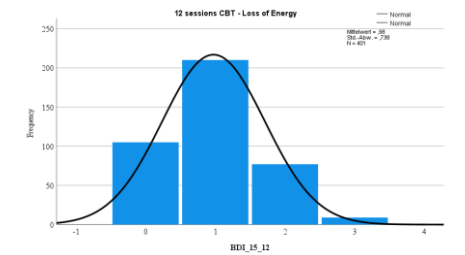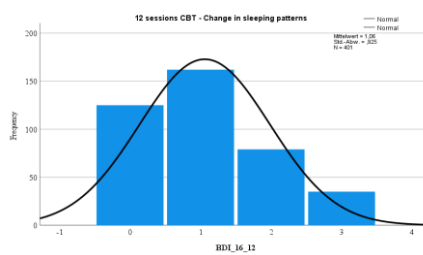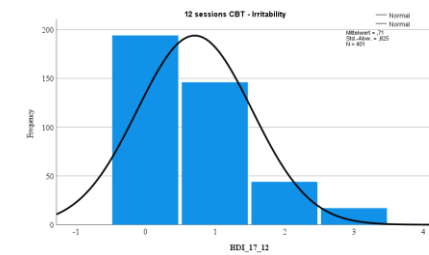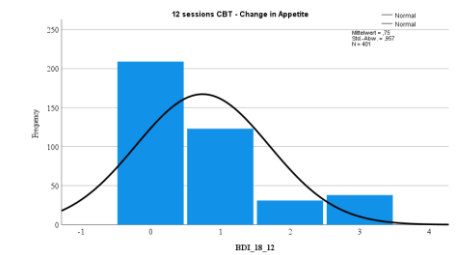

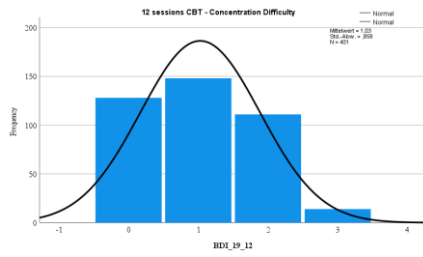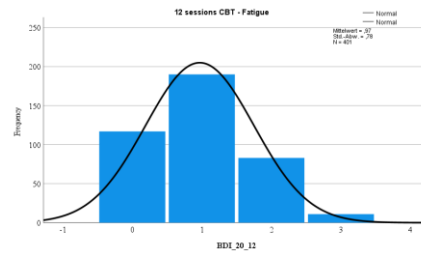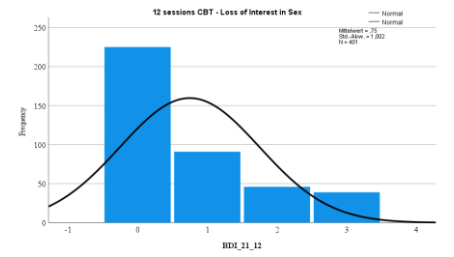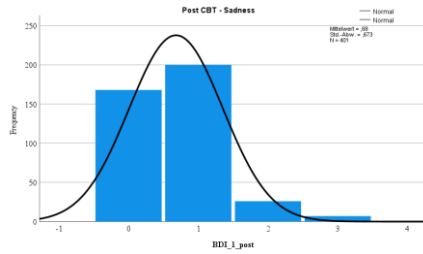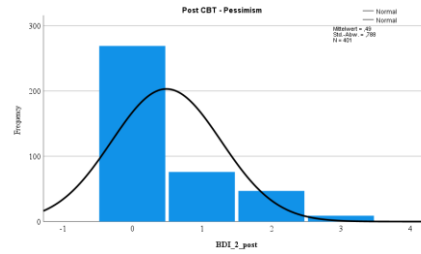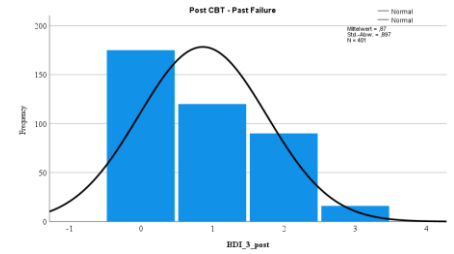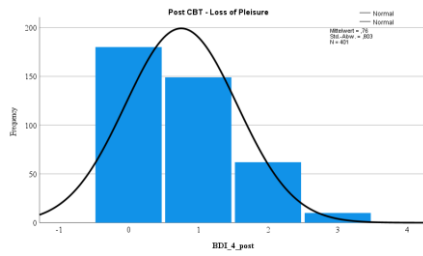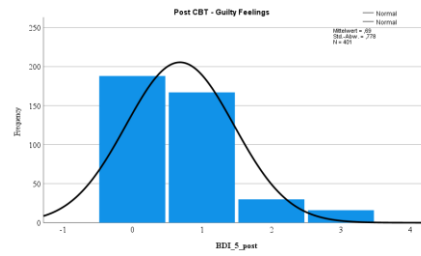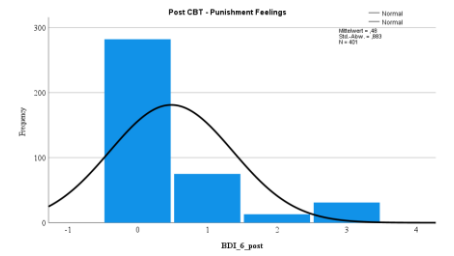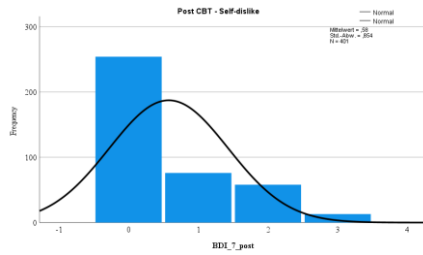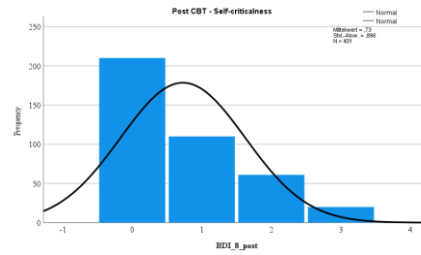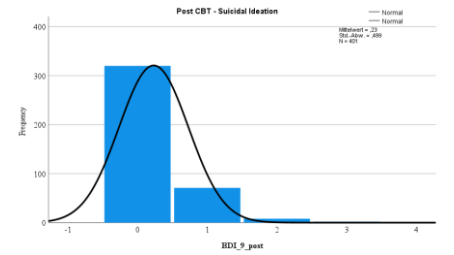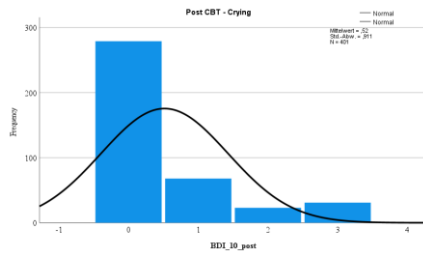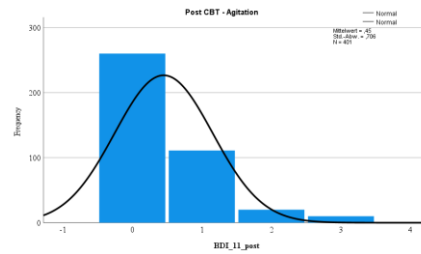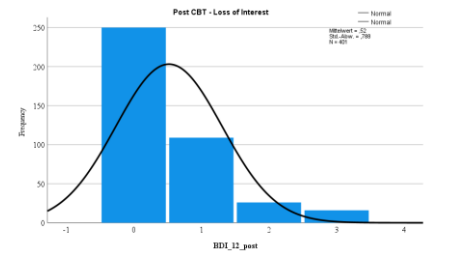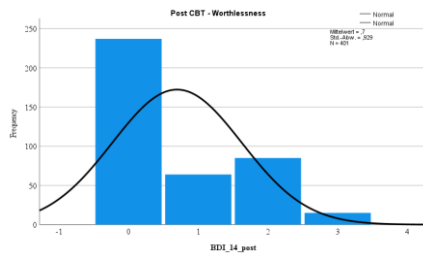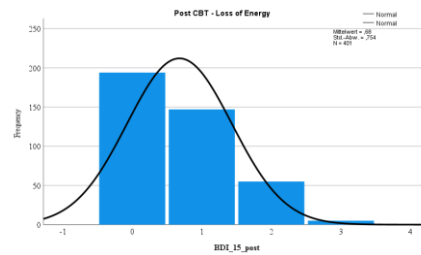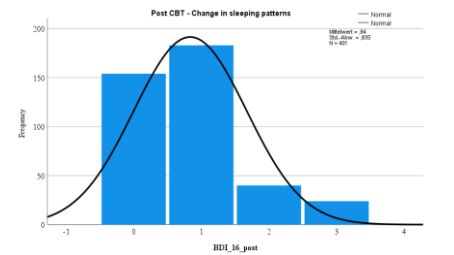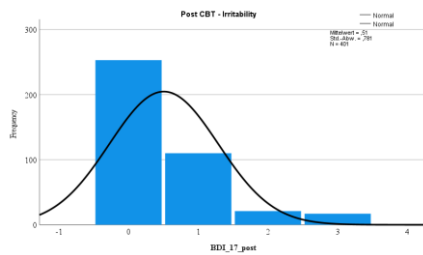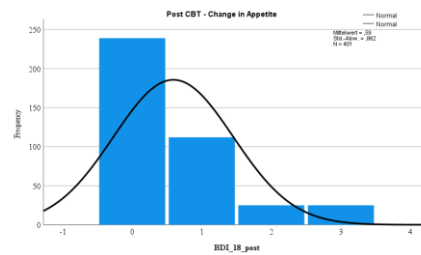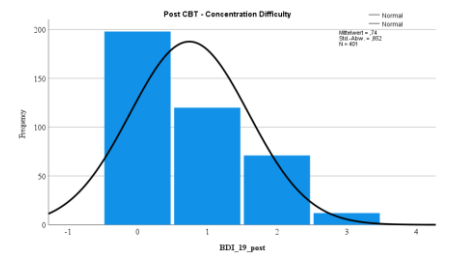

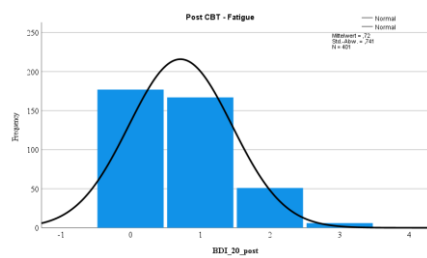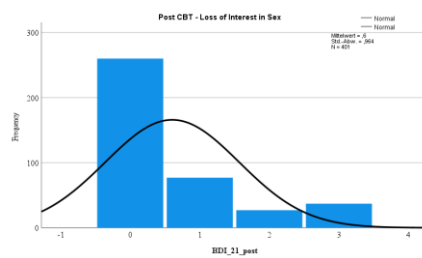

Supplement: Supplementary file 1 [file ijerph-19-07131-s001.zip › ijerph-1724297-supplementary.pdf]
